# Pectin or chitosan coating fortified with eugenol reduces *Campylobacter jejuni* on chicken wingettes and modulates expression of critical survival genes

**DOI:** 10.3382/ps/pey505

**Published:** 2018-11-08

**Authors:** B R Wagle, A Upadhyay, S Shrestha, K Arsi, I Upadhyaya, A M Donoghue, D J Donoghue

**Affiliations:** 1 Department of Poultry Science, University of Arkansas, Fayetteville, AR 72701, USA; 2 Poultry Production and Product Safety Research Unit, ARS, USDA, Fayetteville, AR 72701, USA

**Keywords:** antimicrobial coating, eugenol, *Campylobacter*, gene expression, postharvest poultry

## Abstract

*Campylobacter jejuni* infection in humans is strongly associated with the consumption of contaminated poultry products. With increasing consumer demand for minimally processed and natural product, there is a need for novel intervention strategies for controlling *C. jejuni*. Antimicrobial coatings are increasingly being used for preventing food contamination due to their efficacy and continuous protection of product. This study investigated the efficacy of pectin and chitosan coating fortified with eugenol to reduce *C. jejuni* on chicken wingettes. Pectin, chitosan, and eugenol are generally recognized as safe status compounds derived from berries, crustaceans, and cloves respectively. Each wingette was inoculated with a mixture of 4 wild-type strains of *C. jejuni* (approximately 10^7^ CFU/sample) and randomly assigned to controls, pectin (3%), chitosan (2%), eugenol (0.5, 1, or 2%), or their combinations. Following 1 min of coating, wingettes were air-dried, vacuum sealed, and sampled on 0, 1, 3, 5, and 7 d of refrigerated storage for *C. jejuni* and aerobic counts (n = 5 wingettes/treatment/d). In addition, the effect of treatments on wingette color and expression of *C. jejuni* survival/virulence genes was evaluated. All 3 doses of eugenol or chitosan significantly reduced *C. jejuni* and aerobic bacteria from 0 d through 7 d. Incorporation of 2% eugenol in chitosan improved coating efficiency and reduced *C. jejuni* counts by approximately 3 Log CFU/sample at the end of 7 d of storage (*P* < 0.05). Similarly, the antimicrobial efficacy of pectin was improved by 2% eugenol and the coating reduced *C. jejuni* by approximately 2 Log CFU/sample at 7 d of storage. Chitosan coating with 2% eugenol also showed greater reductions of total aerobic counts as compared to individual treatments of eugenol and chitosan. No significant difference in the color of chicken wingettes was observed between treatments. Exposure of *C. jejuni* to eugenol, chitosan, or combination significantly modulated select genes encoding for motility, quorum sensing, and stress response. Results demonstrate the potential of pectin or chitosan coating fortified with eugenol as a postharvest intervention against *C. jejuni* contamination on poultry products.

## INTRODUCTION

*Campylobacter* is a major foodborne pathogen causing bacterial illness in humans worldwide (Mangen et al., 2016; Marder et al., 2017). The incidence of this pathogen recently surpassed the incidence of *Salmonella* (17.43 vs. 16.66 per 100,000) in the United States with the incorporation of culture-independent diagnostic tests (Marder et al., 2017). Out of 17 species of *Campylobacter, Campylobacter jejuni* is responsible for 90% of the campylobacteriosis in humans (Hermans et al., 2011). *C. jejuni* is frequently associated with gastroenteritis, reactive arthritis, and Guillain-Barré syndrome (Spiller, 2007; Gradel et al., 2009). The primary source of human *Campylobacter* infection reported through risk assessment studies is the consumption and handling of poultry products (Friedman et al., 2004; Danis et al., 2009). The high level of *Campylobacter* in the ceca of birds (approximately 10^8^ CFU/g) and low infective dose (approximately 500 CFU) poses a serious public health concern if carcasses are not properly decontaminated (Beery et al., 1988; Black et al., 1988; Achen et al., 1998).

Studies have shown that *C. jejuni* survives during poultry processing and can cross-contaminate poultry carcasses (Stern et al., 2001; Allen et al., 2007). The poultry producers rely on the use of various chemicals for washing poultry carcasses to decrease the microbial load. Peracetic acid is the most commonly used antimicrobial for decontamination of carcasses during processing; however, it results in minimal reduction (approximately 1.5 Log) and can cause irritation at high concentration (>1,000 ppm) that could lead to occupational hazards (Bauermeister et al., 2008; Nagel et al., 2013; Pechacek et al., 2015; The Poultry Site, 2015). Similarly, chlorine has limited effectiveness and its efficacy further decreases in the presence of organic matter and a pH above 7.0 (Northcutt et al., 2005; Oyarzabal, 2005). The generation of potential mutagens from the reaction of chlorine and organic materials further raises concerns owing to associated health hazards, including cancer (Donato and Zani, 2010; Dore, 2015). As an alternative to peractic acid and chlorine, various other chemicals including trisodium phosphate, hydrogen peroxide, and organic acids have been studied (Zhao and Doyle, 2006; Bauermeister et al., 2008; Riedel et al., 2009; Birk et al., 2010). However, the aforementioned chemicals have limited effectiveness and can cause discoloration of carcass and residues in meat (Bilgili et al., 1998; SCVPH, 1998; EFSA BIOHAZ Panel, 2014).

Numerous studies have focused on plant-derived antimicrobials as an alternative of conventional chemical-based treatments to decontaminate food products (Pei et al., 2009; Mattson et al., 2011; Olaimat et al., 2014; Calo et al., 2015; Olaimat and Holley, 2015; Upadhyay et al., 2015, 2016; Woo-Ming, 2015; Wagle et al., 2017a). The antimicrobial coating on poultry products represents a viable intervention to reduce or eliminate foodborne pathogens (Cagri et al., 2004; Ricke and Hanning, 2013). However, few studies have utilized antimicrobial coating on poultry cuts to reduce *Campylobacter* (Olaimat et al., 2014; Woo-Ming, 2015), and there are no reports on the efficacy of pectin and chitosan coating fortified with eugenol in reducing *C. jejuni* load on chicken wingettes. The incorporation of antimicrobial agents in the coatings offers several advantages such as increased contact time, and possible synergism between 2 compounds thereby requiring low concentrations to inhibit or reduce foodborne pathogens (Cagri et al., 2004; Sangsuwan et al., 2009). Additionally, the coatings remain on the food product thereby protecting foods from contamination during storage and handling. Pectin is a plant-derived heteropolysaccharide and commonly used as a gelling and thickening agent in jelly, marmalades, and confectionaries and as edible coating in foods (Moalemiyan et al., 2012). Chitosan is a linear polysaccharide obtained from crustaceans with significant antimicrobial activity against *Salmonella, Listeria*, and *C. jejuni* (Ganan et al., 2009; Olaimat et al., 2014; Olaimat and Holley, 2015; Upadhyay et al., 2015; Woo-Ming, 2015).

Various active components with significant antimicrobial efficacy from plant sources have been reported (Burt, 2004; Calo et al., 2015). Eugenol (EG) is the active component of clove oil (*Eugenia caryophyllus*) and has shown significant antimicrobial efficacy as an antimicrobial wash or as chitosan-based coating on food products against various foodborne pathogens including *Listeria monocytogenes* (Upadhyay et al., 2015), *Salmonella* (Mattson et al., 2011; Upadhyaya et al., 2016), and *Escherichia coli* (Pei et al., 2009). All of the aforementioned compounds are classified as generally recognized as safe by the US FDA for use in foods (Code of Federal Regulations 21 part 184, 170, and 172 for pectin, chitosan, and eugenol, respectively).

The objective of this study was to investigate the efficacy of pectin and chitosan coating fortified with eugenol to reduce *C. jejuni* on chicken wingettes. In addition, the effect of treatments on the color of chicken wingettes was evaluated. Moreover, the effect of treatments on the expression of *C. jejuni* genes essential for survival and virulence was also determined.

## MATERIALS AND METHODS

### *Campylobac*ter Strains and Culture Conditions

Four wild-type strains (S-1, S-3, S-4, S-8) of *C. jejuni* isolated from commercial poultry were cultured according to a standard published method (Wagle et al., 2017a,b). Each *C. jejuni* strain was cultured in *Campylobacter* enrichment broth (**CEB**; catalog no. 7526A, Neogen Corp., Lansing, MI) for 48 h followed by subculture for 24 h under microaerophilic conditions at 42°C. The strains were centrifuged at 3,000 rpm for 10 min and washed twice in Butterfield's phosphate diluent (**BPD**; 0.625 mM potassium dihydrogen phosphate, pH 7.2). Each strain was appropriately diluted for plating, and equal portions of the strains were combined to use as the inoculum for the study.

### Preparation of Coating Treatments

Two coating materials namely pectin and chitosan were used as carriers of eugenol on chicken wingettes. For the study with pectin, 3% pectin solution was prepared as described previously (Upadhyaya et al., 2016). Briefly, 3 g of pectin powder obtained from citrus peel (catalog no. P9135, Sigma-Aldrich Co., St. Louis, MO) was added to BPD and heated to 60°C for 15 min. For the study with chitosan, medium molecular weight (**MMW**; 190 to 310 kDa) chitosan (catalog no. 448877, Sigma-Aldrich) was chosen as carrier for eugenol and its 2% solution was prepared in 50 mM acetic acid (catalog no. UN2789, Thermo Fisher Scientific, Fair Lawn, NJ) according to a previously published method with slight modifications (Upadhyay et al., 2015). Three concentrations of eugenol (0.5, 1, and 2%) were prepared by adding required volume of eugenol (catalog no. E51791, Sigma-Aldrich) into BPD solution followed by mixing with a magnetic stirrer for 30 min. The eugenol coating treatments (0.5, 1, and 2% EG) were prepared by adding appropriate quantity of eugenol in 3% pectin or 2% chitosan solution. The concentrations of coating treatments were selected based on preliminary experiments on adherence and antimicrobial strength of the coating on chicken wingettes. The BPD and 50 mM acetic acid in BPD were included as controls. The pHs of all the solutions were adjusted using 10 N NaOH to 6.5 ± 0.2.

### Evaluation of Antimicrobial Efficacy of Coating Treatments on Chicken Wingettes

A published method was used to evaluate the antimicrobial activity of coating treatments (Olaimat et al., 2014). Briefly, chicken wingettes were made from chicken wings procured from University of Arkansas pilot processing plant and inoculated with 50 μL mixture of *C. jejuni* (approximately 10^7^ CFU/sample) followed by air-drying for 30 min to facilitate bacterial adherence. Wingettes were coated with controls (BPD, acetic acid), eugenol (0.5, 1, and 2%), coating materials (3% pectin or 2% chitosan), or coating materials fortified with eugenol for 1 min followed by air-drying for 30 min on each side. All the samples were vacuum-sealed and stored at 4°C until sampling on 0, 1, 3, 5, and 7 d.

### Enumeration of *C. jejun*i and Aerobic Bacteria on Chicken Wingettes

For the processing of chicken wingettes, each wingette was dipped in 30 mL of Dey-Engley neutralizing broth (catalog no. C7371, Hardy Diagnostics, Santa Maria, CA) and blended using stomacher (Stomacher^®^ 400 Circulator, Steward Ltd, Worthing, West Sussex, UK) at 250 rpm for 30 s. For all samples, a serial dilution (1:10) of each sample was made and plated on *Campylobacter* line agar plates (Line, 2001) and tryptic soy agar (catalog no. DF0369-17-6, Becton, Dickinson and Company, Sparks, MD) plates. *C. jejuni* counts were enumerated after incubation at 42°C under microaerophilic condition for 48 h and aerobic bacteria were enumerated after incubation under aerobic condition at 37°C for 24 h.

### Color Analysis

For the color analysis, a separate batch of chicken wingettes not inoculated with *C. jejuni* was allocated and coated with treatments as mentioned above. Following air-drying of wingette samples, color of the samples was measured using a chroma meter (CR 400/410, Konica Minolta Sensing Americas, Inc., Ramsey, NJ) as described previously (Wagle et al., 2017a). The chroma meter provides information about 3 different colors (L, a, and b indicating relative lightness, redness, and yellowness, respectively). The instrument was calibrated against a tile, and average color values were recorded from 3 different locations on each sample. The samples were then vacuum-sealed to store at 4°C.

### Gene Expression Analysis Using Real-Time Quantitative PCR

The effect of eugenol, chitosan, and their combination on the expression of survival and virulence genes of *C. jejuni* was studied as described previously (Wagle et al, 2017a,b) using real-time quantitative PCR (**RT-qPCR**) in the presence of chicken meat exudate. Chicken meat exudate was prepared according to a standard published method (Birk et al., 2004). Following the mid-log growth of a wild strain of *C. jejuni* (S-8) in CEB at 42°C under microaerophilic condition, bacteria were exposed to chicken meat exudate treated with subinhibitory concentrations (**SICs**) of eugenol (0.0125%) or chitosan (0.0125%) or their combination for 1 h at 25°C. The total RNA was extracted using RNA mini kit (catalog no. 12183018A, Invitrogen, Carlsbad, CA) and treated with DNase I (catalog no. 18068015, Thermo Fisher Scientific, Foster city, CA). The complementary DNAs (**cDNA**) were prepared using iScript cDNA synthesis kit (catalog no. 1708890, Bio-Rad Laboratories, Inc., Hercules, CA). Primer 3 software (National Center for Biotechnology Information, Bethesda, MD) was used for designing all the primers from Gene Bank and obtained from Integrated DNA Technologies, Inc. (Coralville, IA) (Table 1). The amplified products were detected by using SYBR Green reagent (iQ SYBR Green Supermix, catalog no. 1708880, Bio-Rad Laboratories, Inc.). Data were normalized to endogenous control (16S rRNA), and relative quantification of amplified genes was calculated using the comparative critical threshold (ΔΔCt) method on the QuantStudio 3 Real-Time PCR system (Applied Biosystems, Thermo Fisher). Duplicate samples were used, and the study was repeated 3 times (n = 6).

**Table 1.** Primers used for gene expression analysis using real-time quantitative PCR.

Gene with accession no.	Primer	Sequence (5′-3′)
16S-rRNA (NC_002163.1)	Forward	5′- ATAAGCACCGGCTAACTCCG-3′
(product length 78 bp)	Reverse	5′-TTACGCCCAGTGATTCCGAG-3′
*motA* (NC_002163.1)	Forward	5′-AGCGGGTATTTCAGGTGCTT-3′
(product length 75 bp)	Reverse	5′-CCCCAAGGAGCAAAAAGTGC-3′
*motB* (NC_002163.1)	Forward	5′-AATGCCCAGAATGTCCAGCA-3′
(product length 51 bp)	Reverse	5′-AGTCTGCATAAGGCACAGCC-3′
*fliA* (NC_002163.1)	Forward	5′-AGCTTTCACGCCGTTACGAT-3′
(product length 56 bp)	Reverse	5′-TCTTGCAAAACCCCAGAAGT-3′
*cetA* (NC_002163.1)	Forward	5′-CCTACCATGCTCTCCTGCAC-3′
(product length 78 bp)	Reverse	5′-CGCGATATAGCCGATCAAACC-3′
*cetB* (NC_002163.1)	Forward	5′-GCCTTGTTGCTGTTCTGCTC-3′
(product length 88 bp)	Reverse	5′-TTCCGTTCGTCGTATGCCAA -3′
*cadF* (NC_002163.1)	Forward	5′-CGCGGGTGTAAAATTCCGTC-3′
(product length 135 bp)	Reverse	5′-TCCTTTTTGCCACCAAAACCA-3′
*ciaB* (NC_002163.1)	Forward	5′-TCTCAGCTCAAGTCGTTCCA-3′
(product length 50 bp)	Reverse	5′-GCCCGCCTTAGAACTTACAA-3′
*jlpA* (NC_002163.1)	Forward	5′-AGCACACAGGGAATCGACAG-3′
(product length 66 bp)	Reverse	5′-TAACGCTTCTGTGGCGTCTT-3′
*sodB* (NC_002163.1)	Forward	5′-CAAAACTTCAAATGGGGGCGT-3′
(product length 98 bp)	Reverse	5′-CACAGCCACAGCCTGTACTT-3′
*katA* (NC_002163.1)	Forward	5′-ATGCTGAACGCGATGTGAGA -3′
(product length 99 bp)	Reverse	5′- CGCGGATGAAGAATGTCGGA-3′
*luxS* (NC_002163.1)	Forward	5′-AGTGTTGCAAAAGCTTGGGA-3′
(product length 106 bp)	Reverse	5′-GCATTGCACAAGTTCCGCAT-3′
*racS* (NC_002163.1)	Forward	5′-AGACAAGTTGCCGAAGTTGC -3′
(product length 79 bp)	Reverse	5′-AGGCGATCTTGCCTACTTCA-3′
*racR* (NC_002163.1)	Forward	5′-AGAGAACAGCTTGTAAGTCGCT-3′
(product length 83 bp)	Reverse	5′-ACCCTTAAGCGACCGATGAT -3′

### Statistical Analysis

The study was a completely randomized design. In total, 4 trials were conducted on the chicken wingettes with 5 wingettes per treatment per storage day for 225 and 250 wingettes in total per pectin and chitosan trial, respectively. Each trial was replicated twice (n = 950 chicken wingettes per total trials). For the analysis, bacterial counts were logarithmic transferred to maintain the homogeneity of variance (Byrd et al., 2001) and the data of color analysis and gene expression were pooled before analysis. The data were analyzed by using the PROC MIXED procedure in SAS version 9.3 software (SAS Institute Inc., Cary, NC). The treatment means were separated by least-square means analysis, and the significance level was *P* < 0.05 for statistical difference.

## RESULTS

### Antimicrobial Efficacy of Coating Treatment With or Without Eugenol Against C. jejuni on Chicken Wingettes

The effect of eugenol in reducing *C. jejuni* on chicken wingettes was evaluated in both pectin and chitosan trials presented in Tables 2 and 3, respectively. *C. jejuni* counts recovered from the wingettes not subjected to coating treatment (baseline) ranged from 5.27 to 6.72 Log CFU/sample in all the trials. Washing with BPD produced a maximum reduction of approximately 1.44 Log CFU/sample compared to baseline in pectin trials (Table 2); however, in the chitosan trial 2 (Table 3), the reduction was not significant on 0 and 3 d. The 0.5, 1, and 2% eugenol consistently reduced *C. jejuni* counts on chicken wingettes by at least 0.8, 0.56, 0.66, and 0.72 Log CFU/sample compared to BPD control in trials 1 and 2 of pectin and chitosan studies, respectively. Among the 3 doses of eugenol, 2% produced significantly greater reductions than 0.5% on 0 and 7 d in both pectin trials, 7 d in chitosan trial 1, and on 0 and 1 d in chitosan trial 2. Similarly, 2% EG was more effective than 1% EG on 0 and 7 d in pectin trial 1, 1 and 3 d in pectin trial 2, 7 d in chitosan trial 1, and 0 and 1 d in chitosan trial 2. There was significant difference in anti-*Campylobacter* effect between 0.5 and 1% EG on 7 d in pectin trial 2; however, the results were not consistent between trials.

**Table 2.** The efficacy of eugenol (0, 0.5, 1, or 2%), pectin (0 or 3%), and their combinations as coating treatment to reduce *C. jejuni* on chicken wingettes.^1^

Trial no.	Treatments	0 d	1 d	3 d	5 d	7 d
1	Baseline	6.55 ± 0.07^a,x^	6.14 ± 0.14^a,x,y^	5.77 ± 0.11^a,y^	6.06 ± 0.05^a,y^	5.81 ± 0.05^a,y^
	BPD control	5.41 ± 0.05^b,x^	5.21 ± 0.15^b,x,y^	5.03 ± 0.06^b,x,y^	4.98 ± 0.07^b,x,y^	4.83 ± 0.11^b,y^
	0.5% Eugenol	4.57 ± 0.21^c,x^	4.04 ± 0.11^c,d,y^	3.98 ± 0.17^c,y^	3.98 ± 0.19^c,y^	3.58 ± 0.11^c,d,y^
	1% Eugenol	4.51 ± 0.11^c,x^	4.01 ± 0.11^c,d,y^	3.94 ± 0.12^c,d,y,z^	3.98 ± 0.13^c,y^	3.29 ± 0.39^d,z^
	2% Eugenol	3.95 ± 0.12^d,x^	3.88 ± 0.09^c,d,e,x^	3.60 ± 0.14^c,d,x^	3.55 ± 0.27^c,d,x^	2.58 ± 0.37^e,y^
	3% Pectin	5.57 ± 0.12^b,x^	5.35 ± 0.18^b,x,y^	4.79 ± 0.11^b,z^	5.03 ± 0.17^b,y,z^	4.82 ± 0.23^b,z^
	0.5% Eugenol +3% Pectin	4.66 ± 0.16^c,x^	4.31 ± 0.08^c,x,y^	3.97 ± 0.11^c,d,y^	3.81 ± 0.12^c,z^	3.83 ± 0.07^c,z^
	1% Eugenol + 3% Pectin	4.38 ± 0.13^c,d,x^	3.75 ± 0.14^d,e,y^	3.73 ± 0.10^c,d,y^	3.86 ± 0.20^c,y^	3.60 ± 0.23^c,d,y^
	2% Eugenol + 3% Pectin	3.40 ± 0.27^e,x^	3.50 ± 0.27^e,x^	3.50 ± 0.11^d,x^	3.16 ± 0.30^d,x^	3.17 ± 0.18^d,x^
2	Baseline	6.72 ± 0.05^a,w^	6.11 ± 0.06^a,x^	6.17 ± 0.16^a,x^	5.63 ± 0.06^a,y^	5.27 ± 0.05^a,z^
	BPD control	5.69 ± 0.07^b,w^	4.92 ± 0.05^c,x^	4.73 ± 0.05^b,x,y^	4.53 ± 0.08^c,x,y^	4.51 ± 0.08^b,y^
	0.5% Eugenol	4.74 ± 0.13^c,d,x^	4.19 ± 0.17^d,e,y^	3.93 ± 0.08^c,d,y^	3.85 ± 0.16^d,e,y^	3.95 ± 0.08^c,y^
	1% Eugenol	4.42 ± 0.23^d,e,f,x^	4.37 ± 0.07^d,x^	4.11 ± 0.05^c,x^	3.48 ± 0.17^e,y^	3.48 ± 0.26^d,y^
	2% Eugenol	4.12 ± 0.27^f,x^	3.75 ± 0.15^e,x,y^	3.62 ± 0.22^d,y^	3.46 ± 0.18^e,y^	3.31 ± 0.24^d,y^
	3% Pectin	5.55 ± 0.14^b,w^	5.49 ± 0.17^b,x,w^	5.06 ± 0.05^b,x,y^	5.03 ± 0.11^b,y^	4.55 ± 0.14^b,z^
	0.5% Eugenol +3% Pectin	4.90 ± 0.13^c,w^	4.31 ± 0.37^d,x^	4.07 ± 0.12^c,d,x,y^	4.05 ± 0.14^d,x,y^	3.73 ± 0.19^c,d,y^
	1% Eugenol + 3% Pectin	4.61 ± 0.18^c,d,e,x^	4.41 ± 0.16^d,x^	4.20 ± 0.06^c,x,y^	3.83 ± 0.19^d,e,y^	3.57 ± 0.12^c,d,y^
	2% Eugenol + 3% Pectin	4.22 ± 0.33^e,f,w^	3.85 ± 0.17^e,w,x^	3.65 ± 0.13^d,x,y^	3.47 ± 0.15^e,x,y^	3.37 ± 0.23^d,y^

^1^n = 5 replicates per treatment per day per trial. Values (Log CFU/sample) presented as mean ± standard error of the mean. Within the same trial, different superscripts a–f in columns and w–z in rows differ significantly at *P* < 0.05.

**Table 3.** The efficacy of eugenol (0, 0.5, 1, or 2%), chitosan (0 or 2%), and their combinations as coating treatment to reduce *C. jejuni* on chicken wingettes.^1^

Trial no.	Treatments	0 d	1 d	3 d	5 d	7 d
1	Baseline	6.09 ± 0.08^a,x^	5.78 ± 0.09^a,x^	5.85 ± 0.11^a,x^	5.89 ± 0.11^a,x^	5.93 ± 0.12^a,x^
	BPD control	5.08 ± 0.03^b,x^	4.98 ± 0.12^b,x^	4.88 ± 0.15^b,x^	4.90 ± 0.10^b,x^	4.82 ± 0.08^b,x^
	0.5% Eugenol	4.28 ± 0.09^c,x^	4.30 ± 0.10^c,d,x^	4.07 ± 0.06^c,x^	4.20 ± 0.06^c,x^	4.16 ± 0.09^c,x^
	1% Eugenol	3.88 ± 0.41^c,d,x^	4.24 ± 0.08^c,d,x^	3.95 ± 0.23^c,x^	4.05 ± 0.15^c,x^	4.15 ± 0.08^c,x^
	2% Eugenol	3.81 ± 0.07^c,d,x^	3.92 ± 0.27^c,d,e,x^	3.67 ± 0.21^c,x^	3.56 ± 0.32^c,x^	2.61 ± 0.51^d,y^
	Acetic acid control	6.00 ± 0.12^a,x^	4.81 ± 0.06^b,y^	5.07 ± 0.08^b,y^	4.97 ± 0.05^b,y^	4.83 ± 0.12^b,y^
	2% Chitosan	3.94 ± 0.10^c,d,x^	3.82 ± 0.27^d,e,f,x^	3.95 ± 0.07^c,x^	3.97 ± 0.14^c,x^	3.71 ± 0.14^c,x^
	0.5% Eugenol +2% Chitosan	3.50 ± 0.16^d,e,x^	3.24 ± 0.29^e,f,g,x,y^	3.66 ± 0.20^c,x^	3.83 ± 0.31^c,x^	2.75 ± 0.61^d,y^
	1% Eugenol + 2% Chitosan	2.93 ± 0.17^e,y^	3.16 ± 0.24^f,g,x,y^	3.85 ± 0.39^c,x^	3.53 ± 0.08^c,x,y^	2.89 ± 0.58^d,y^
	2% Eugenol + 2% Chitosan	2.91 ± 0.20^e,x^	2.86 ± 0.25 ^g,x^	2.85 ± 0.58^d,x^	2.66 ± 0.50^d,x^	2.45 ± 0.48^d,x^
2	Baseline	6.40 ± 0.03^a,x^	6.11 ± 0.06^a,x,y^	5.32 ± 0.22^a,z^	5.62 ± 0.10^a,y^	5.87 ± 0.02^a,x,y,z^
	BPD control	5.69 ± 0.09^a,x^	4.98 ± 0.06^b,x,y^	4.78 ± 0.10^a,y^	4.70 ± 0.09^b,y^	5.02 ± 0.07^b,x,y^
	0.5% Eugenol	4.32 ± 0.31^b,x^	4.11 ± 0.05^c,x^	4.01 ± 0.15^b,x^	3.98 ± 0.10^c,x^	3.83 ± 0.26^c,d,x^
	1% Eugenol	4.36 ± 0.16^b,x^	3.80 ± 0.27^c,x^	4.01 ± 0.08^b,x^	3.95 ± 0.08^c,x^	3.81 ± 0.31^c,d,x^
	2% Eugenol	3.34 ± 0.25^c,d,x,y^	2.99 ± 0.23^d,e,y^	3.30 ± 0.28^b,c,d,x,y^	3.78 ± 0.11^c,d,x^	3.22 ± 0.67^d,e,x,y^
	Acetic acid control	5.79 ± 0.07^a,x^	5.00 ± 0.07^b,y^	4.79 ± 0.09^a,y^	5.00 ± 0.09^a,b,y^	5.16 ± 0.06^a,b,x,y^
	2% Chitosan	4.30 ± 0.09^b,x^	3.93 ± 0.16^c,x^	4.01 ± 0.25^b,x^	4.07 ± 0.21^c,x^	4.20 ± 0.08^c,x^
	0.5% Eugenol +2% Chitosan	3.95 ± 0.19^b,c,x^	3.55 ± 0.45^c,d,x^	3.89 ± 0.15^b,c,x^	3.76 ± 0.37^c,d,x^	3.87 ± 0.33^c,d,x^
	1% Eugenol + 2% Chitosan	3.11 ± 0.58^d,x^	3.44 ± 0.30^c,d,x^	3.26 ± 0.20^c,d,x^	3.14 ± 0.35^d,e,x^	3.05 ± 0.62^e,x^
	2% Eugenol + 2% Chitosan	2.62 ± 0.54^d,x^	2.70 ± 0.20^e,x^	3.05 ± 0.18^d,x^	2.73 ± 0.50^e,x^	3.03 ± 0.28^e,x^

^1^n = 5 replicates per treatment per day per trial. Values (Log CFU/sample) presented as mean ± standard error of the mean. Within the same trial, different superscripts a–g in columns and x–z in rows differ significantly at *P* < 0.05.

The effect of pectin as a coating material and the eugenol–pectin coating combinations against *C. jejuni* is presented in Table 2. Pectin consistently reduced *C. jejuni* counts on majority of storage time points by at least 0.6 Log CFU/sample as compared to non-coated (baseline) chicken wingettes. However, there were no consistent differences between pectin and BPD controls in both trials. Incorporation of eugenol in the pectin coating consistently improved the anti-*Campylobacter* activity of pectin (*P* < 0.05), but the combination was similar in efficacy as compared with eugenol in majority of storage days (*P* > 0.05). Among the 3 combination treatments, 2% eugenol–pectin combination produced at least 2.1 Log CFU/sample reduction and this was significantly greater when compared with 0.5% combinations in most of the storage days (0, 1, 5, and 7 d in trial 1 and 0, 1, and 5 d in trial 2). Similarly, this reduction was significantly greater in 2 storage days (0 and 5 d in trial 1 and on 1 and 3 d in trial 2) as compared to 1% combination treatment. There were no significant differences between 0.5 and 1% combination treatments of pectin and eugenol with the exception on 1 d in trial 1.

Table 3 shows the effect of chitosan coating either alone or in combination with eugenol against *C. jejuni* on chicken wingettes. Chitosan coating consistently reduced *C. jejuni* counts with a range from 0.74 to 2.06 Log CFU/sample in both trials. Acetic acid (control for chitosan coating with or without eugenol) reduced *C. jejuni* counts in trial 1 from 1 to 7 d; however, in trial 2, an inconsistent reduction was observed as compared to baseline. When compared with BPD control, acetic acid did not significantly reduce *C. jejuni* counts on majority of storage days. The combination of eugenol and chitosan produced consistent reduction of at least 0.9 Log CFU/sample when compared with acetic acid in both trials. Among the 3 combinations of eugenol and chitosan when compared with acetic acid control, the maximum reduction was approximately 3 Log CFU/sample on 0 d of both trials observed with 1 and 2% eugenol–chitosan combinations. The 2% eugenol–chitosan coating produced greater reduction of *C. jejuni* counts than that by chitosan alone in both trials (*P* < 0.05). This reduction was also significantly different from 2% eugenol alone in majority of storage days (0, 1, 3, and 5 d) in trial 1 and only on 5 d in trial 2. Additionally, there were no consistent differences among 0.5, 1, and 2% eugenol–chitosan combinations.

### Antimicrobial Efficacy of Coating Treatment With or Without Eugenol Against Aerobic Bacteria on Chicken Wingettes

Tables 4 and 5 show the effect of eugenol on the total aerobic bacterial counts on chicken wingettes (from pectin and chitosan study, respectively). The aerobic counts on chicken wingettes not subjected to any treatment (baseline) were approximately 4.5 Log CFU/sample on 0 d. The aerobic counts increased by at least 1.2 Log CFU/sample by the end of 7 d in all trials (*P* < 0.05). The treatment with BPD failed to reduce aerobic bacteria in all the trials except on 0 and 1 d in chitosan trial 1 (Table 5). All the tested doses of eugenol consistently reduced aerobic counts by at least 0.51 Log CFU/sample from 0 to 7 d when compared with BPD controls. There was no significant difference among 0.5, 1, and 2% eugenol treatments on majority of days (3, 5, and 7 d) in both pectin trials and chitosan trial 2 and on 0 and 1 d in chitosan trial 1.

**Table 4.** The efficacy of eugenol (0, 0.5, 1, or 2%), pectin (0 or 3%), and their combinations as coating treatment against aerobic bacteria on chicken wingettes.^1^

Trial no.	Treatments	0 d	1 d	3 d	5 d	7 d
1	Baseline	4.69 ± 0.03^a,z^	4.73 ± 0.13^a,z^	5.50 ± 0.18^a,y^	6.65 ± 0.05^a,x^	7.15 ± 0.10^a,w^
	BPD control	4.39 ± 0.07^a,z^	4.43 ± 0.17^a,z^	5.23 ± 0.20^a,y^	6.46 ± 0.21^a,x^	6.98 ± 0.06^a,w^
	0.5% Eugenol	3.86 ± 0.16^b,y^	3.32 ± 0.24^c,d,z^	4.03 ± 0.11^c,d,y^	5.26 ± 0.08^c,x^	6.29 ± 0.07^b,c,w^
	1% Eugenol	3.73 ± 0.09^b,c,z^	3.69 ± 0.12^c,z^	3.71 ± 0.22^c,d,z^	5.03 ± 0.16^c,d,y^	6.34 ± 0.08^b,c,x^
	2% Eugenol	3.42 ± 0.07^c,d,z^	3.66 ± 0.16^c,d,z^	3.71 ± 0.14^c,d,z^	5.22 ± 0.10^c,y^	6.16 ± 0.13^b,c,x^
	3% Pectin	4.35 ± 0.11^a,z^	4.43 ± 0.18^a,z^	4.73 ± 0.18^b,z^	5.99 ± 0.12^b,y^	6.53 ± 0.22^b,x^
	0.5% Eugenol +3% Pectin	3.55 ± 0.13^b,c,d,z^	3.47 ± 0.14^c,d,z^	3.86 ± 0.12^c,d,z^	5.29 ± 0.08^c,y^	6.26 ± 0.10^b,c,x^
	1% Eugenol + 3% Pectin	3.42 ± 0.10^c,d,z^	3.62 ± 0.15^c,d,z^	3.81 ± 0.08^c,d,z^	4.71 ± 0.13^d,y^	6.36 ± 0.05^b,c,x^
	2% Eugenol + 3% Pectin	3.29 ± 0.18^d,z^	3.27 ± 0.22^d,z^	3.57 ± 0.16^d,z^	4.19 ± 0.10^e,y^	5.98 ± 0.19^c,x^
2	Baseline	4.56 ± 0.08^a,z^	5.32 ± 0.12^a,y^	5.99 ± 0.17^a,x^	7.05 ± 0.19^a,w^	7.05 ± 0.12^a,w^
	BPD control	4.35 ± 0.14^a,z^	5.25 ± 0.18^a,y^	6.16 ± 0.14^a,x^	6.95 ± 0.10^a,w^	7.04 ± 0.14^a,w^
	0.5% Eugenol	3.81 ± 0.06^b,z^	4.41 ± 0.20^b,y^	5.00 ± 0.13^c,x^	5.98 ± 0.09^c,d,w^	6.19 ± 0.06^b,c,w^
	1% Eugenol	3.68 ± 0.23^b,z^	4.15 ± 0.28^b,c,y^	5.02 ± 0.13^b,c,x^	5.67 ± 0.22^d,w^	6.35 ± 0.02^b,c,v^
	2% Eugenol	3.68 ± 0.11^b,y^	3.34 ± 0.19^d,y^	5.05 ± 0.14^b,c,x^	5.77 ± 0.12^c,d,w^	6.04 ± 0.09^c,w^
	3% Pectin	4.26 ± 0.24^a,y^	5.58 ± 0.10^a,x^	5.43 ± 0.26^b,x^	6.42 ± 0.03^b,w^	6.53 ± 0.21^b,w^
	0.5% Eugenol +3% Pectin	3.84 ± 0.06^b,y^	4.01 ± 0.06^b,c,y^	4.99 ± 0.18^c,x^	6.18 ± 0.06^b,c,w^	6.26 ± 0.06^b,c,w^
	1% Eugenol + 3% Pectin	3.55 ± 0.11^b,y^	3.80 ± 0.10^c,y^	4.96 ± 0.08^c,x^	5.77 ± 0.11^c,d,w^	6.18 ± 0.05^b,c,w^
	2% Eugenol + 3% Pectin	2.98 ± 0.37^c,y^	3.35 ± 0.32^d,y^	4.76 ± 0.15^c,x^	5.74 ± 0.07^d,w^	5.96 ± 0.12^c,w^

^1^n = 5 replicates per treatment per day per trial. Values (Log CFU/sample) presented as mean ± standard error of the mean. Within the same trial, different superscripts a–e in columns and v–z in rows differ significantly at *P* < 0.05.

**Table 5.** The effect of eugenol (0, 0.5, 1, or 2%), chitosan (0 or 2%), and their combinations as coating treatment against aerobic bacteria on chicken wingettes.^1^

Trial no.	Treatments	0 d	1 d	3 d	5 d	7 d
1	Baseline	4.30 ± 0.10^a,z^	4.84 ± 0.08^a,y^	5.59 ± 0.15^a,w,x^	5.85 ± 0.06^a,w^	5.54 ± 0.06^a,b,x^
	BPD control	3.93 ± 0.07^b,z^	4.63 ± 0.11^b,y^	5.46 ± 0.10^a,b,x^	5.85 ± 0.04^a,w^	5.42 ± 0.16^b,x^
	0.5% Eugenol	3.62 ± 0.10^c,z^	4.09 ± 0.08^c,y^	4.59 ± 0.07^c,x,y^	5.33 ± 0.02^b,w^	4.81 ± 0.09^c,d,x^
	1% Eugenol	3.28 ± 0.11^d,z^	3.99 ± 0.09^c,y^	4.58 ± 0.05^c,y^	5.35 ± 0.03^b,w^	5.00 ± 0.11^c,x^
	2% Eugenol	3.32 ± 0.18^c,d,y^	3.94 ± 0.09^c,x^	4.04 ± 0.16^d,x^	4.93 ± 0.09^c,w^	4.68 ± 0.06^d,w^
	Acetic acid control	3.96 ± 0.07^b,z^	4.46 ± 0.06^a,b,y^	5.28 ± 0.14^b,x^	5.74 ± 0.11^a,w^	5.76 ± 0.06^a,w^
	2% Chitosan	3.10 ± 0.09^d,y^	3.95 ± 0.10^c,x^	4.85 ± 0.13^c,w^	4.87 ± 0.12^c,w^	4.71 ± 0.04^c,d,w^
	0.5% Eugenol +2% Chitosan	2.73 ± 0.06^e,z^	3.51 ± 0.06^d,y^	4.20 ± 0.10^d,x^	4.81 ± 0.05^c,w^	4.59 ± 0.02^d,w^
	1% Eugenol + 2% Chitosan	2.53 ± 0.19^e,y^	3.83 ± 0.05^c,x^	4.08 ± 0.25^d,x^	4.63 ± 0.09^c,w^	4.53 ± 0.05^d,w^
	2% Eugenol + 2% Chitosan	2.61 ± 0.18^e,z^	3.23 ± 0.15^d,y^	3.54 ± 0.15^e,x^	3.96 ± 0.09^d,w^	3.94 ± 0.13^e,w^
2	Baseline	4.79 ± 0.20^a,y^	5.38 ± 0.03^a,x^	5.52 ± 0.05^a,x^	5.77 ± 0.02^a,x^	6.61 ± 0.05^a,w^
	BPD control	4.50 ± 0.11^a,y^	5.31 ± 0.21^a,x^	5.34 ± 0.06^a,x^	5.63 ± 0.03^a,x^	6.49 ± 0.08^a,w^
	0.5% Eugenol	3.74 ± 0.10^b,c,y^	4.79 ± 0.11^b,x^	4.83 ± 0.07^b,x^	5.01 ± 0.08^b,x^	5.65 ± 0.04^b,w^
	1% Eugenol	3.99 ± 0.17^b,y^	4.22 ± 0.24^c,d,y^	4.75 ± 0.07^b,c,x^	4.97 ± 0.12^b,w^	5.58 ± 0.06^b,w^
	2% Eugenol	3.71 ± 0.13^b,c,y^	3.73 ± 0.16^e,y^	4.53 ± 0.08^b,c,x^	4.96 ± 0.06^b,x^	5.45 ± 0.17^b,w^
	Acetic acid control	4.58 ± 0.16^a,y^	5.34 ± 0.12^a,x^	5.31 ± 0.17^a,x^	5.60 ± 0.05^a,x^	6.26 ± 0.17^a,w^
	2% Chitosan	3.42 ± 0.16^e,d,z^	4.52 ± 0.14^b,c,x^	4.51 ± 0.11^b,c,x^	4.75 ± 0.05^b,w,x^	5.21 ± 0.14^b,w^
	0.5% Eugenol +2% Chitosan	3.14 ± 0.14^d,e y^	4.75 ± 0.23^b,x^	4.70 ± 0.15^b,c,x^	4.95 ± 0.08^b,w,x^	5.34 ± 0.16^b,w^
	1% Eugenol + 2% Chitosan	2.92 ± 0.22^e,z^	3.98 ± 0.24^d,e,y^	4.31 ± 0.13^c,d,y^	4.80 ± 0.19^b,x^	5.33 ± 0.32^b,w^
	2% Eugenol + 2% Chitosan	2.90 ± 0.17^e,y^	2.79 ± 0.51^f,y^	3.94 ± 0.10^d,x^	4.15 ± 0.18^c,x^	4.71 ± 0.32^c,w^

^1^n = 5 replicates per treatment per day per trial. Values (Log CFU/sample) presented as mean ± standard error of the mean. Within the same trial, different superscripts a–e in columns and w–z in rows differ significantly at *P* < 0.05.

Table 4 also shows the effect of pectin and eugenol–pectin coating on the total aerobic counts on chicken wingettes. Pectin coating significantly reduced aerobic counts starting from 3 to 7 d in both trials. Eugenol–pectin combination coating also reduced the counts consistently and improved the antibacterial efficacy of pectin at the beginning (0 to 5 d in trial 1 and 0 to 3 d in trial 2) but not on 7 d in both trials. However, no significant differences have been observed among 0.5, 1, and 2% eugenol–pectin combinations on majority of storage days (0, 1, 3, 7 d) in trial 1, and on 3 and 7 d in trial 2.

The effect of chitosan and eugenol–chitosan combination on the total aerobic counts on chicken wingettes is shown in Table 5. Treatment of chicken wingettes with acetic acid did not significantly reduce aerobic counts with the exception on 0 and 3 d in trial 1. In contrast, chitosan coating significantly reduced the counts across all storage days in both trials. This reduction ranged from 0.72 to 1.2 and from 0.86 to 1.37 Log CFU/sample in trials 1 and 2, respectively. Similar result was observed with 0.5 and 1% eugenol–chitosan combination coating. The 2% eugenol–-chitosan coating showed greater reductions as compared to 0.5% combination treatments beginning from 3 d in both trials whereas in comparison with 1% combination treatments, it was significantly different on 1, 3, 5, and 7 d in trial 1 and on 1, 5, and 7 d in trial 2. Moreover, the reduction obtained with 2% eugenol–chitosan was significantly different from the individual's treatment of chitosan and eugenol across all storage days in both trials with the exception on 0 d in trial 2.

### Effect of Coating Treatments on the Color of Chicken Wingettes

The coating of chicken wingettes with eugenol, pectin, chitosan, and their combination did not affect the lightness, redness, and yellowness of meat (Supplementary Tables S1 and 2). However, the refrigerated storage time had a significant effect on the yellowness of chicken wingettes and decreased the color value by at least 3 units in all the treatments (Supplementary Table S1C). Similarly, storage time significantly increased the lightness of chicken wingettes treated with chitosan alone or in combination with 0.5 or 1% EG by 3 units (Supplementary Table S2A).

### Effect of Eugenol and Chitosan on Expression of C. jejuni Virulence Genes

The gene expression profile of *C. jejuni* in response to SICs of eugenol, chitosan, and their combinations is shown in Table 6. The presence of SICs of eugenol, chitosan, and their combination significantly changed the expression of select genes coding for pathogen motility, stress response, quorum sensing, and attachment to epithelial cells. The SIC of eugenol significantly downregulated the expression of genes coding for motility (*motA, motB*), stress response (*katA*), and quorum sensing (*luxS*). However, energy taxis genes (*cetA, cetB*) responsible for directional motility, attachment genes (*cadF, ciaB, jlpA*), and 2-component regulatory proteins (RacR-RacS) were not affected (*P* > 0.05). Chitosan at SIC level, downregulated *motA* gene, however, upregulated select genes for motility (*motB*), attachment (*ciaB, jlpA*), and stress response (*sodB*). Other genes essential for *C. jejuni* motility (*fliA, cetA, cetB*), stress response (*katA*), quorum sensing (*luxS*), and 2-component regulatory system (*racS*-*racR*) were not changed by chitosan (*P* > 0.05). Similar to chitosan, the eugenol–chitosan combination downregulated *motA* gene and upregulated genes *motB, ciaB, jlpA*, and *sodB*. In addition, the combination also downregulated genes *luxS* and *katA*, an effect similar to eugenol treatment. The combination of eugenol and chitosan reduced the expression of *cetA* as compared to control (*P* < 0.05). The individual treatments did not modulate the expression of *cetA* (*P* > 0.05). The acetic acid treatment did not affect the expression of tested genes (*P* > 0.05).

**Table 6.** The effect of 0.0125% MMW chitosan and 0.0125% eugenol on the expression of *C. jejuni* genes essential for survival and virulence.

Gene	Gene product function	Relative gene expression (Log_10_ RQ)^1^				
		Treatments				
		Control	Acetic acid	Chitosan	Eugenol	Eugenol + chitosan
*motA*	Motility	0^a^	–0.09 ± 0.03^a^	–0.35 ± 0.17^b^	–0.51 ± 0.15^b^	–0.32 ± 0.11^b^
*motB*	Motility	0^b^	0.13 ± 0.09^b^	0.21 ± 0.03^a^	–0.13 ± 0.06^c^	0.24 ± 0.08^a^
*fliA*	Motility	0^a^	0.09 ± 0.04^a^	0.08 ± 0.09^a^	–0.04 ± 0.02^a^	0.13 ± 0.10^a^
*cetA*	Energy taxis protein/motility	0^b^	0.08 ± 0.14^b^	0.13 ± 0.07^b^	0.05 ± 0.06^b^	0.20 ± 0.08^a^
*cetB*	Energy taxis protein/motility	0^a^	–0.05 ± 0.14^a^	–0.05 ± 0.29^a^	–0.02 ± 0.10^a^	0.08 ± 0.36^a^
*cadF*	Attachment	0^a^	0.11 ± 0.06^a^	0.08 ± 0.09^a^	–0.10 ± 0.04^a^	0.10 ± 0.09^a^
*ciaB*	Attachment	0^b^	0.18 ± 0.16^a,b^	0.25 ± 0.02^a^	–0.02 ± 0.08^b^	0.32 ± 0.10^a^
*jlpA*	Attachment	0^c^	0.09 ± 0.06^c^	0.43 ± 0.08^b^	0.08 ± 0.15^c^	0.55 ± 0.03^a^
*sodB*	Superoxide dismutase	0^b^	–0.03 ± 0.04^b^	0.20 ± 0.04^a^	0.08 ± 0.10^b^	0.19 ± 0.04^a^
*katA*	Catalase/oxidative stress	0^a^	–0.01 ± 0.05^a^	0.14 ± 0.12^a^	–0.23 ± 0.11^b^	–0.18 ± 0.06^b^
*luxS*	Quorum sensing	0^a^	0.05 ± 0.11^a^	0.09 ± 0.06^a^	–0.29 ± 0.11^b^	–0.23 ± 0.08^b^
*racS*	Two-component sensor/histidine kinase	0^a^	0.06 ± 0.12^a^	0.09 ± 0.21^a^	–0.06 ± 0.07^a^	0.05 ± 0.30^a^
*racR*	Two-component regulator	0^a^	0.14 ± 0.16^a^	0.06 ± 0.27^a^	–0.11 ± 0.08^a^	–0.02 ± 0.06^a^

^1^n = 6 replicates per treatment. Values (mean ± standard error of the mean) with different superscripts within a row indicate significant change in gene expression (*P* < 0.05).

## DISCUSSION

*Campylobacter* contamination of poultry product is one of the major risk factors for human campylobacteriosis (Friedman et al., 2004). Despite rigorous search for interventions to be utilized in the processing facility, the pathogen is widely present on raw poultry products (Stern et al., 2001). In addition, there is an increase in consumer preference toward product with minimal processing and chemical treatment. A potential strategy for controlling *Campylobacter* is by antimicrobial coating of raw poultry products. In this study, we investigated the efficacy of eugenol as a coating treatment of chicken wingettes and hypothesized that increasing contact time between compounds and bacteria could improve the antibacterial activity of eugenol.

In order to coat the chicken wingettes with eugenol, we selected 2 coating materials, pectin and chitosan, which are extensively studied as films in the food industry as an alternative of conventional packaging materials (Aider, 2010; Moalemiyan et al., 2012). Pectin dissolves at neutral pH, whereas chitosan requires acidification. Therefore, we used acetic acid at 50 mM to dissolve the compound. Pectin itself did not exhibit antimicrobial activity against *C. jejuni* (Table 2). Similar findings were reported previously where pectin coating did not significantly reduce coliforms on shrimp (Alvarez et al., 2014) and *Salmonella* on eggs (Upadhyaya et al., 2016). In contrast to pectin, coating of chicken wingettes with MMW chitosan exerted significant antimicrobial activity against *C. jejuni* (Table 3). Olaimat et al. (2014) used chitosan/κ-carrageenan combination coating on chicken breast. They found significant reduction (up to 2.78 Log CFU/g) of *C. jejuni* with the coating containing mustard extract. In the present study, the incorporation of select concentrations of eugenol in coating materials significantly improved the efficacy of pectin and chitosan coating materials. This finding was also similar to previous reports from other studies where the incorporation of eugenol significantly improved the efficacy of pectin coating against *Salmonella* Enteritidis (Upadhyaya et al., 2016) and chitosan coating against *L. monocytogenes* (Upadhyay et al., 2015). However, the pectin fortified with eugenol was similar in efficacy as compared with eugenol alone. Since pectin coating will be present on the poultry products during storage, it would potentially protect the product from microbial contamination during handling or subsequent processing until it reaches the consumer. In addition, coatings could control moisture transfer, gas exchange, or oxidation processes thereby protecting the foods (Rojas-Graü et al., 2009). Therefore, although found to be similar in its antimicrobial efficacy in the current experimental design, eugenol–pectin coating provides additional advantages than eugenol wash.

Reducing aerobic counts on chicken wingettes is important to increase the shelf life of product during refrigeration (Kim and Marshall, 2000). Coating of raw chicken wingettes with eugenol and its coating materials significantly reduced total aerobic counts (Tables 4 and 5). However, none of the treatments checked further growth of aerobic bacteria on chicken wingettes with storage days. Kim and Marshall (2000) had similar findings when 1% organic acids treated chicken wings were stored at 4°C for 12 d. Since chicken skin harbors diverse bacteria including psychrophiles (Cox et al., 1998; Kim et al., 2017), the increase in aerobic plate count could be due to growth of these bacteria. Even though there was an increase in aerobic plate counts by at least 1.2 Log CFU/sample on 7 d in controls, the counts in controls as well as in treatments were below the critical point (8 Log CFU/cm^2^) where fresh meat produces sliminess due to bacterial spoilage (Cox et al., 1998).

We investigated the effect of the treatments on color of chicken wingettes since it is one of the key factors to assess the quality of poultry products for purchaser. We observed that there were no significant differences in color (lightness, redness, yellowness) of chicken wingettes between treatments and controls (Supplementary Tables). Khan et al. (2015) had also observed similar results with 0.05% eugenol on raw chicken. During storage, studies have shown that changes in color values are more pronounced within 6 h after post-mortem and become less variable later on (Petracci and Fletcher, 2002). It was also reported previously that color of poultry meat changed to lighter and more brownish with time due to growth of microbes, pH, lipid oxidation, and other deteriorating factors (Khan et al., 2015). We did not observe any significant change in color except yellowness with storage days probably due to the effect of coating material.

Previous studies from our lab (Arambel et al., 2015; Upadhyay et al., 2017; Wagle et al., 2017a,b) as well as other researchers (Castillo et al., 2014; Oh and Jeon, 2015; Kovács et al., 2016) have determined that phytochemicals at subinhibitory concentrations modulate the expression of genes in various microbes including *C. jejuni*. We investigated the effect of SICs of eugenol and chitosan on the expression of *C. jejuni* genes associated with survival and virulence to delineate their potential mechanism of action. Since pectin failed to reduce *C. jejuni* counts compared to BPD controls (Table 2), its effect on *C. jejuni* gene expression was not determined. Gene expression analysis was studied in the presence of chicken meat juice to represent the meat environment, especially because chicken meat juice is known to modulate the physiology of *C. jejuni* thereby enhancing their survival in the poultry products (Birk et al., 2004; Brown et al., 2014). Several researchers have used 16S rRNA as an endogenous control in real-time qPCR (Klančnik et al., 2006; Tasara and Stephan, 2007; Hays, 2009; Koolman et al., 2016), and we used the same gene for calibrating the expression of other genes. A variety of genes responsible for bacterial virulence have been characterized for *C. jejuni* (Hermans et al., 2011). The movement of *C. jejuni* toward substrate at low temperature (4°C) is responsible for their survival in meat (Hazeleger et al., 1998). The motility of *C. jejuni* is imparted through flagella and encoded by the genes *motA, motB*, and *fliA*, which also play a role in the pathogenesis of human *Campylobacter* infection (Young et al., 2007). In addition, the energy taxis genes (*cetA, cetB*) are essential for motility in response to stimuli, attachment, and biofilm formation on various surfaces (Kalmokoff et al., 2006; Hermans et al., 2011). Moreover, *cadF* and *jlpA* are responsible for cell surface attachment (Jin et al., 2003; Hermans et al., 2011). The 2-component regulatory proteins (RacR-RacS) are necessary for temperature-dependent growth of *C. jejuni* (Hermans et al., 2011). Previously, it was shown that *C. jejuni luxS* mutants were unable to survive in meat environment (Ligowska et al., 2011). Similarly, stress response (*katA, sodB*) genes are important for adaptation and survival of *C. jejuni* (Atack and Kelly, 2009). We observed that eugenol significantly downregulated the expression of select genes encoding motility (*motA, motB*) and quorum sensing (*luxS*) in *C. jejuni* thereby potentially limiting the survival in meat environment (Table 6). Similarly, eugenol also downregulated *katA* gene, which is in contrast to the previous reports (Kovács et al., 2016) with clove oils possibly due to difference in *C. jejuni* strains (wild type vs. NCTC 11168). The expression level of majority of genes in eugenol–chitosan combination was similar to that of either eugenol or chitosan except *cetA* and *jlpA*. We observed an upregulation of few virulence genes (*motB, ciaB, jlpA*) and stress gene (*sodB*) in response to chitosan. This could potentially be due to trigger of a compensatory or stress response pathway. A transcriptomic study would shed more light on the effect of chitosan on virulence and other critical genes. Overall, these findings suggest that the aforementioned treatments could affect the potential of *C. jejuni* to survive in meat and cause disease in humans.

## CONCLUSION

Pectin and chitosan coating fortified with eugenol on the poultry cuts consistently reduce *C. jejuni*. In addition, eugenol, chitosan, and their combination modulated transcription of several genes essential for survival and virulence of *C. jejuni*. Since a 2-Log reduction of *C. jejuni* from poultry carcass translates into more than 90% reduction in the risk of human *Campylobacter* infections (Nauta et al., 2016), the aforementioned treatments represent a safe, effective, and natural approach that could improve poultry product safety. Follow-up studies testing the effect of the coating on the organoleptic properties of meat are warranted.

## SUPPLEMENTARY DATA

**Supplementary Table S1**. The effect of eugenol (0, 0.5, 1, or 2%), pectin (0 or 3%), and their combinations as coating treatment on the color (A: lightness, B: redness, C: yellowness) of chicken wingettes.

**Supplementary Table S2**. The effect of eugenol (0, 0.5, 1, or 2%), chitosan (0 or 2%), and their combinations as coating treatment on the color (A: lightness, B: redness, C: yellowness) of chicken wingettes.

Supplemental Tables
