# Supplementary material for: Pectin or chitosan coating fortified with eugenol reduces Campylobacter jejuni on chicken wingettes and modulates expression of critical survival genes
Source: Poult Sci. 2018 Nov 8;98(3):1461–71. doi: 10.3382/ps/pey505 (PMC6377438; doi:10.3382/ps/pey505)
Supplement: Supplemental Tables [file pey505_supplemental_tables.zip › ps-18-08057-S001.docx]

**Supplementary table 1:** The effect of eugenol (0, 0.5, 1 or 2%), pectin (0 or 3%) and their combinations as coating treatment on the color (Table 1A: lightness, 1B: redness, 1C: yellowness) of chicken wingettes^1^

**Table 1A**

|  | **0 d** | **1 d** | **3 d** | **5 d** | **7 d** |
| --- | --- | --- | --- | --- | --- |
| Baseline | 69.62±0.64^a,x^ | 70.30±0.58^a,x^ | 71.03±0.76^a,x^ | 71.25±0.77^a,x^ | 71.06±0.50^a,x^ |
| BPD control | 70.36±1.00^a,x^ | 70.57±1.12^a,x^ | 70.96±0.85^a,x^ | 71.21±0.81^a,x^ | 71.26±0.66^a,x^ |
| 0.5% Eugenol | 71.52±0.76^a,x^ | 70.28±0.59^a,x^ | 71.08±0.60^a,x^ | 70.54±0.65^a,x^ | 70.45±0.71^a,x^ |
| 1% Eugenol | 71.41±0.29^a,x^ | 71.23±0.45^a,x^ | 71.24±0.53^a,x^ | 71.57±0.46^a,x^ | 71.37±0.47^a,x^ |
| 2% Eugenol | 71.54±0.73^a,x^ | 71.50±0.42^a,x^ | 71.92±0.44^a,x^ | 71.22±0.62^a,x^ | 71.82±0.57^a,x^ |
|  |  |  |  |  |  |
| 3% Pectin | 69.63±1.05^a,x^ | 70.02±0.86^a,x^ | 71.05±0.50^a,x^ | 70.80±0.69^a,x^ | 70.42±0.56^a,x^ |
| 0.5% Eugenol +3% Pectin | 70.11±0.70^a,x^ | 69.62±0.65^a,x^ | 70.13±0.41^a,x^ | 70.93±0.60^a,x^ | 71.12±0.35^a,x^ |
| 1% Eugenol + 3% Pectin | 70.33±0.87^a,x^ | 70.44±0.40^a,x^ | 70.95±0.71^a,x^ | 71.77±0.64^a,x^ | 72.12±0.64^a,x^ |
| 2% Eugenol + 3% Pectin | 70.15±0.76^a,x^ | 69.77±0.81^a,x^ | 70.35±0.99^a,x^ | 70.29±0.90^a,x^ | 70.75±0.96^a,x^ |

^1^Color values presented as mean ± standard error of the mean. Different superscript a-c in columns and x-z in rows differ significantly at P<0.05.

**Table 1B**

|  | **0 d** | **1 d** | **3 d** | **5 d** | **7 d** |
| --- | --- | --- | --- | --- | --- |
| Baseline | 4.03±0.37^a,x^ | 3.15±0.52^a,x^ | 3.28±0.40^a,x^ | 3.28±0.34^a,x^ | 3.08±0.31^a,x^ |
| BPD control | 3.74±0.47^a,x^ | 3.23±0.35^a,x^ | 3.37±0.50^a,x^ | 3.54±0.50^a,x^ | 3.26±0.41^a,x^ |
| 0.5% Eugenol | 4.29±0.20^a,x^ | 3.17±0.12^a,x^ | 3.33±0.18^a,x^ | 3.33±0.22^a,x^ | 3.18±0.21^a,x^ |
| 1% Eugenol | 3.23±0.48^a,x^ | 2.42±0.34^a,x^ | 2.17±0.42^a,x^ | 2.41±0.34^a,x^ | 2.52±0.35^a,x^ |
| 2% Eugenol | 3.53±0.45^a,x^ | 3.41±0.52^a,x^ | 3.30±0.40^a,x^ | 2.91±0.43^a,x^ | 2.67±0.48^a,x^ |
|  |  |  |  |  |  |
| 3% Pectin | 3.48±0.32^a,x^ | 3.09±0.63^a,x^ | 3.30±0.55^a,x^ | 2.83±0.40^a,x^ | 2.85±0.52^a,x^ |
| 0.5% Eugenol +3% Pectin | 3.80±0.61^a,x^ | 3.16±0.67^a,x^ | 3.02±0.68^a,x^ | 2.85±0.31^a,x^ | 2.97±0.29^a,x^ |
| 1% Eugenol + 3% Pectin | 3.25±0.32^a,x^ | 2.70±0.24^a,x^ | 2.40±0.42^a,x^ | 2.49±0.17^a,x^ | 2.13±0.12^a,x^ |
| 2% Eugenol + 3% Pectin | 3.59±0.46^a,x^ | 2.96±0.37^a,x^ | 3.11±0.35^a,x^ | 2.83±0.39^a,x^ | 2.65±0.23^a,x^ |

^1^Color values presented as mean ± standard error of the mean. Different superscript a-c in columns and x-z in rows differ significantly at P<0.05.

**Table 1C**

|  | **0 d** | **1 d** | **3 d** | **5 d** | **7 d** |
| --- | --- | --- | --- | --- | --- |
| Baseline | 12.22±0.83^a,x^ | 8.76±0.83^a,y^ | 7.42±0.93^a,y^ | 7.95±0.80^a,y^ | 7.05±0.85^a,y^ |
| BPD control | 11.48±0.50^a,x^ | 8.11±1.14^a,y^ | 7.22±1.00^a,y^ | 7.40±1.01^a,y^ | 7.41±0.99^a,y^ |
| 0.5% Eugenol | 10.86±0.87^a,x^ | 7.63±1.28^a,y^ | 7.37±1.50^a,y^ | 7.20±1.49^a,y^ | 7.74±1.64^a,y^ |
| 1% Eugenol | 12.65±0.71^a,x^ | 9.45±1.19^a,y^ | 9.31±1.40^a,y^ | 8.93±1.36^a,y^ | 8.85±0.97^a,y^ |
| 2% Eugenol | 10.80±1.20^a,x^ | 8.28±0.86^a,xy^ | 7.60±0.90^a,y^ | 6.82±1.25^a,y^ | 7.75±1.03^a,y^ |
|  |  |  |  |  |  |
| 3% Pectin | 13.39±0.68^a,x^ | 10.15±0.94^a,y^ | 9.71±0.64^a,y^ | 8.80±0.70^a,y^ | 8.77±0.47^a,y^ |
| 0.5% Eugenol +3% Pectin | 12.67±0.87^a,x^ | 9.06±1.35^a,y^ | 7.90±1.26^a,y^ | 7.64±1.30^a,y^ | 7.19±1.50^a,y^ |
| 1% Eugenol + 3% Pectin | 12.21±0.46^a,x^ | 8.78±0.38^a,y^ | 7.51±0.22^a,y^ | 7.58±0.37^a,y^ | 7.67±0.37^a,y^ |
| 2% Eugenol + 3% Pectin | 11.33±0.47^a,x^ | 7.70±0.66^a,y^ | 7.74±0.61^a,y^ | 6.45±0.65^a,y^ | 6.56±0.60^a,y^ |

^1^Color values presented as mean ± standard error of the mean. Different superscript a-c in columns and x-z in rows differ significantly at P<0.05.
