# Supplementary material for: Pectin or chitosan coating fortified with eugenol reduces Campylobacter jejuni on chicken wingettes and modulates expression of critical survival genes
Source: Poult Sci. 2018 Nov 8;98(3):1461–71. doi: 10.3382/ps/pey505 (PMC6377438; doi:10.3382/ps/pey505)
Supplement: Supplemental Tables [file pey505_supplemental_tables.zip › ps-18-08057-S002.docx]

**Supplementary Table 2:** The effect of eugenol (0, 0.5, 1 or 2%), chitosan (0 or 2%) and their combinations as coating treatment on the color (Table 2A: lightness, 2B: redness, 2C: yellowness) of chicken wingettes^1^

**Table 2A**

|  | **0 d** | **1 d** | **3 d** | **5 d** | **7 d** |
| --- | --- | --- | --- | --- | --- |
| Baseline | 72.04±0.55^ab, x^ | 72.94±0.84^a, x^ | 72.99±0.67^a, x^ | 72.66±0.67^ab, x^ | 73.02±0.63^abc, x^ |
| BPD control | 72.21±0.68^ab, x^ | 73.72±0.90^a, x^ | 74.03±0.72^a, x^ | 73.97±0.60^ab, x^ | 74.31±1.17^ab, x^ |
| 0.5% Eugenol | 71.79±0.91^ab, x^ | 72.55±1.17^a, x^ | 72.96±0.97^a, x^ | 72.05±1.08^b, x^ | 71.65±0.96^c, x^ |
| 1% Eugenol | 71.19±0.72^b, y^ | 72.04±1.38^a, xy^ | 72.93±0.73^a, xy^ | 73.73±0.49^ab, y^ | 72.01±1.28^c, xy^ |
| 2% Eugenol | 72.49±0.69^ab, x^ | 73.58±0.45^a, x^ | 74.62±0.67^a, x^ | 73.47±0.67^ab, x^ | 75.24±0.67^ab, x^ |
|  |  |  |  |  |  |
| Acetic acid control | 73.46±0.80^a, xy^ | 73.18±0.77^a, xy^ | 74.92±0.18^a, x^ | 74.34±0.46^a, xy^ | 72.20±0.96^bc, y^ |
| 2% Chitosan | 72.63±0.40^ab, y^ | 73.33±0.75^a, xy^ | 74.34±0.64^a, xy^ | 74.47±0.47^a, xy^ | 76.01±0.24^a, x^ |
| 0.5% Eugenol +2% Chitosan | 71.49±0.77^ab, y^ | 72.09±0.36^a, y^ | 74.64±0.43^a, x^ | 73.06±0.92^ab, xy^ | 74.24±0.59^ab, x^ |
| 1% Eugenol + 2% Chitosan | 71.60±0.48^ab, y^ | 72.27±0.63^a, xy^ | 73.69±0.44^a, xy^ | 74.27±0.32^a, x^ | 74.38±0.65^a, x^ |
| 2% Eugenol + 2% Chitosan | 72.13±0.67^ab, x^ | 72.59±1.13^a, x^ | 74.08±0.92^a, x^ | 73.34±0.61^ab, x^ | 73.24±0.94^abc, x^ |

^1^Color values presented as mean ± standard error of the mean. Different superscript a-c in columns and x-z in rows differ significantly at P<0.05.

**Table 2B**

|  | **0 d** | **1 d** | **3 d** | **5 d** | **7 d** |
| --- | --- | --- | --- | --- | --- |
| Baseline | 3.44±0.59^a, y^ | 3.55±0.75^a, xy^ | 3.43±0.41^a, xy^ | 4.04±0.41^a, xy^ | 4.90±0.38^a, x^ |
| BPD control | 3.28±0.49^a, x^ | 3.90±0.72^a, x^ | 3.72±0.86^a, x^ | 3.49±0.42^a, x^ | 4.63±1.09^a, x^ |
| 0.5% Eugenol | 3.75±0.60^a, x^ | 3.88±0.78^a, x^ | 3.32±0.85^a, x^ | 4.22±0.85^a, x^ | 4.47±0.37^a, x^ |
| 1% Eugenol | 3.49±0.80^a, xy^ | 3.52±0.69^a, xy^ | 2.75±0.38^a, y^ | 3.98±0.84^a, xy^ | 4.78±1.12^a, x^ |
| 2% Eugenol | 3.02±0.90^a, x^ | 3.16±0.83^a, x^ | 2.10±0.60^a, x^ | 2.93±0.50^a, x^ | 3.99±0.34^a, x^ |
|  |  |  |  |  |  |
| Acetic acid control | 3.13±0.50^a, y^ | 2.94±0.37^a, y^ | 2.86±0.31^a, y^ | 3.85±0.56^a, xy^ | 5.19±0.68^a, x^ |
| 2% Chitosan | 3.04±0.44^a, x^ | 3.30±0.37^a, x^ | 2.73±0.43^a, x^ | 3.38±0.39^a, x^ | 4.13±0.37^a, x^ |
| 0.5% Eugenol +2% Chitosan | 3.54±0.77^a, xy^ | 4.17±0.66^a, x^ | 2.39±0.41^a, y^ | 3.06±0.52^a, xy^ | 3.95±0.43^a, xy^ |
| 1% Eugenol + 2% Chitosan | 3.85±0.87^a, x^ | 3.25±0.26^a, x^ | 2.53±0.24^a, x^ | 3.31±0.34^a, x^ | 3.88±0.39^a, x^ |
| 2% Eugenol + 2% Chitosan | 2.99±0.29^a, x^ | 2.80±0.35^a, x^ | 2.38±0.27^a, x^ | 2.68±0.34^a, x^ | 3.91±0.40^a, x^ |

^1^Color values presented as mean ± standard error of the mean. Different superscript a-c in columns and x-z in rows differ significantly at P<0.05.

**Table 2C**

|  | **0 d** | **1 d** | **3 d** | **5 d** | **7 d** |
| --- | --- | --- | --- | --- | --- |
| Baseline | 11.69±1.07^a, x^ | 10.08±0.33^ab, x^ | 8.77±1.35^a, xy^ | 7.32±0.91^a, y^ | 6.78±0.32^a, y^ |
| BPD control | 12.60±1.07^a, x^ | 11.52±0.98^ab, xy^ | 9.12±1.30^a, yz^ | 7.91±1.33^a, z^ | 9.66±1.31^a, yz^ |
| 0.5% Eugenol | 11.60±0.88^a, x^ | 11.30±1.45^ab, xy^ | 9.72±0.79^a, xy^ | 8.78±0.90^a, y^ | 9.03±1.33^a, y^ |
| 1% Eugenol | 11.49±0.84^a, x^ | 9.19±0.63^b, xy^ | 8.84±0.94^a, xy^ | 8.50±1.18^a, y^ | 8.09±0.78^a, y^ |
| 2% Eugenol | 12.81±0.75^a, x^ | 11.48±0.72^ab, xy^ | 10.21±0.66^a, xy^ | 9.19±0.78^a, y^ | 9.49±0.44^a, y^ |
|  |  |  |  |  |  |
| Acetic acid control | 11.62±1.03^a, x^ | 10.12±0.98^ab, xy^ | 8.13±1.22^a, y^ | 7.93±0.86^a, y^ | 8.83±1.44^a, y^ |
| 2% Chitosan | 13.89±1.33^a, x^ | 12.67±1.71^a, xy^ | 10.29±1.56^a, yz^ | 9.36±1.16^a, z^ | 10.17±1.77^a, z^ |
| 0.5% Eugenol +2% Chitosan | 13.16±0.36^a, x^ | 11.59±0.51^ab, xy^ | 10.66±0.82^a, xy^ | 9.28±1.08^a, y^ | 9.86±0.74^a, y^ |
| 1% Eugenol + 2% Chitosan | 12.91±0.63^a, x^ | 11.07±1.00^ab, xy^ | 9.59±0.65^a, y^ | 8.57±0.97^a, y^ | 9.12±0.71^a, y^ |
| 2% Eugenol + 2% Chitosan | 12.86±0.50^a, x^ | 11.28±0.89^ab, xy^ | 9.77±0.87^a, yz^ | 7.48±0.83^a, z^ | 8.87±0.80^a, yz^ |

^1^Color values presented as mean ± standard error of the mean. Different superscript a-c in columns and x-z in rows differ significantly at P<0.05.
